# DVsc: An Automated Framework for Efficiently Detecting Viral Infection from Single-cell Transcriptomics Data

**DOI:** 10.1093/gpbjnl/qzad007

**Published:** 2023-12-19

**Authors:** Fei Leng, Song Mei, Xiaolin Zhou, Xuanshi Liu, Yefeng Yuan, Wenjian Xu, Chongyi Hao, Ruolan Guo, Chanjuan Hao, Wei Li, Peng Zhang

**Affiliations:** Beijing Key Laboratory for Genetics of Birth Defects, Beijing Pediatric Research Institute; MOE Key Laboratory of Major Diseases in Children; Rare Disease Center, Beijing Children's Hospital, Capital Medical University, National Center for Children's Health, Beijing 100045, China; Beijing Key Laboratory for Genetics of Birth Defects, Beijing Pediatric Research Institute; MOE Key Laboratory of Major Diseases in Children; Rare Disease Center, Beijing Children's Hospital, Capital Medical University, National Center for Children's Health, Beijing 100045, China; Institute of Biomedical Engineering, University of Toronto, Toronto, M5S 3G9, Canada; Beijing Key Laboratory for Genetics of Birth Defects, Beijing Pediatric Research Institute; MOE Key Laboratory of Major Diseases in Children; Rare Disease Center, Beijing Children's Hospital, Capital Medical University, National Center for Children's Health, Beijing 100045, China; Beijing Key Laboratory for Genetics of Birth Defects, Beijing Pediatric Research Institute; MOE Key Laboratory of Major Diseases in Children; Rare Disease Center, Beijing Children's Hospital, Capital Medical University, National Center for Children's Health, Beijing 100045, China; Beijing Key Laboratory for Genetics of Birth Defects, Beijing Pediatric Research Institute; MOE Key Laboratory of Major Diseases in Children; Rare Disease Center, Beijing Children's Hospital, Capital Medical University, National Center for Children's Health, Beijing 100045, China; Beijing Key Laboratory for Genetics of Birth Defects, Beijing Pediatric Research Institute; MOE Key Laboratory of Major Diseases in Children; Rare Disease Center, Beijing Children's Hospital, Capital Medical University, National Center for Children's Health, Beijing 100045, China; Beijing Key Laboratory for Genetics of Birth Defects, Beijing Pediatric Research Institute; MOE Key Laboratory of Major Diseases in Children; Rare Disease Center, Beijing Children's Hospital, Capital Medical University, National Center for Children's Health, Beijing 100045, China; Beijing Key Laboratory for Genetics of Birth Defects, Beijing Pediatric Research Institute; MOE Key Laboratory of Major Diseases in Children; Rare Disease Center, Beijing Children's Hospital, Capital Medical University, National Center for Children's Health, Beijing 100045, China; Beijing Key Laboratory for Genetics of Birth Defects, Beijing Pediatric Research Institute; MOE Key Laboratory of Major Diseases in Children; Rare Disease Center, Beijing Children's Hospital, Capital Medical University, National Center for Children's Health, Beijing 100045, China; Beijing Key Laboratory for Genetics of Birth Defects, Beijing Pediatric Research Institute; MOE Key Laboratory of Major Diseases in Children; Rare Disease Center, Beijing Children's Hospital, Capital Medical University, National Center for Children's Health, Beijing 100045, China

**Keywords:** Single-cell RNA sequencing, Viral transcriptome, Cellular transcriptome, Viral infection, Quantitative analysis, Bulk RNA sequencing

## Abstract

Single-cell RNA sequencing (scRNA-seq) has emerged as a valuable tool for studying cellular heterogeneity in various fields, particularly in virological research. By studying the viral and cellular transcriptomes, the dynamics of viral infection can be investigated at a single-cell resolution. However, limited studies have been conducted to investigate whether RNA transcripts from clinical samples contain substantial amounts of viral RNAs, and a specific computational framework for efficiently detecting viral reads based on scRNA-seq data has not been developed. Hence, we introduce DVsc, an open-source framework for precise quantitative analysis of viral infection from single-cell transcriptomics data. When applied to approximately 200 diverse clinical samples that were infected by more than 10 different viruses, DVsc demonstrated high accuracy in systematically detecting viral infection across a wide array of cell types. This innovative bioinformatics pipeline could be crucial for addressing the potential effects of surreptitiously invading viruses on certain illnesses, as well as for designing novel medicines to target viruses in specific host cell subsets and evaluating the efficacy of treatment. DVsc supports the FASTQ format as an input and is compatible with multiple single-cell sequencing platforms. Moreover, it could also be applied to sequences from bulk RNA sequencing data. DVsc is available at http://62.234.32.33:5000/DVsc.

## Introduction

Understanding the interactions between a virus and its host cells is crucial for the development of effective treatment approaches for infectious diseases. Numerous investigations have been conducted to assess the host immune response to viral infection by bulk tissue or cell population analysis and further experimental examination [1,2]. The impact of the viral life cycle on the host immune system has been systematically explored from both the virus’s perspective, through the analysis of viral sequences, and the host cell’s perspective, through the analysis of transcription profiles [3,4]. Nevertheless, it has been challenging to use high-throughput sequencing strategies to characterize *in vivo* the full map of host−virus interactions at the viral and cellular levels simultaneously. The effectiveness of viral replication is largely dependent on the host, as viruses adapt to take advantage of the host cell machinery. Therefore, cell heterogeneity is directly connected to the viral replication cycle and infection outcome, and the study of cell heterogeneity during the infection process is of significant interest. Achieving widespread infection in a specific cell type is relatively rare, and this unequal susceptibility to infection can be explained by two non-mutually exclusive hypotheses [5,6]. First, viral heterogeneity, which occurs primarily through cross-species transmission, increases the likelihood of host jumping, resulting in increased diversity of viral populations. Second, various characteristics of host heterogeneity, such as host receptor availability, post-translational modifications (PTMs), and antiviral defense diversity, result in distinct cellular settings that are favorable for virus propagation success in each cell.

Breakthroughs in single-cell genomic technology have presented a chance to address this challenge [7,8], as they represent a novel prospect for identifying specific cellular and molecular features that promote or restrict virus replication. Consequently, new targets for inhibiting viral replication could be identified, thereby contributing to a better understanding of host−virus interactions. Single-cell analyses allow simultaneous mapping of both the host and viral transcriptomes in the same single cell and identification of cell subsets with certain phenotypes, which are vital for understanding of host−virus interactions and have the potential to alter the experimental approach used for viral infection research [9–11]. In particular, single-cell sequencing technologies can capture viral diversity to identify sequence variation in viral quasispecies, examine cellular heterogeneity, and explore the immune response to viral infection in infected cells, thereby allowing a systematic investigation of the impact of cell-to-cell diversity on the outcome of viral infection. Moreover, the ability to examine the same single cell before and after viral infection is ideal, because the cellular state and gene expression change in response to infection [7,12,13].

Thus, the purpose of this study was to develop a framework for exploring viral diversity and cell variability in response to viral replication by using single-cell RNA sequencing (scRNA-seq) and to make this open-source pipeline available to the general public for use. With this pipeline, we were able to simultaneously detect infected cells, uncover the composition of multiple viruses within the cell, and obtain the endogenous expression profiles of host genes within the cell. We believe that the application of this pipeline will profoundly impact research on infection and immunity, particularly based on single-cell transcriptomics data. We have developed both an online version and a local version to cater to different user needs. The online version is suitable for users with smaller datasets and for testing purposes. Users can analyze or test their data using the online version. Users with larger datasets can download the local version and run it on their local servers. The software and user instructions for both the online and local versions can be found at http://62.234.32.33:5000/DVsc.

## Method

### Reference database curation

The human reference genome version GRCh38 (hg38) was downloaded from the Ensembl database (http://asia.ensembl.org/index.html). The human mitochondrial genome was downloaded from the MITOMAP database (https://www.mitomap.org/MITOMAP). To create a broader annotation system for the genomes of the whole universe of viruses, a viral reference database was compiled and annotated from several sources. Viral genome sequences were extracted from viruSITE (release 2021.02) and the NCBI RefSeq database (release 2021.08). The annotations obtained from the RefSeq entries were modified using the data obtained from viruSITE, ViralZone, NCBI Taxonomy, and PubMed. All the data were downloaded, preprocessed, and combined into the database using in-house developed scripts. In total, there were 14,698 viral segments from 11,556 different viruses. The HISAT2 [14] index was subsequently built for the viral reference database in addition to the host reference database.

### Data access, preprocessing, and demultiplexing of the scRNA-seq dataset

Sequence Read Archive (SRA) files were downloaded from the Gene Expression Omnibus (GEO), and FASTQ files were split and extracted using the FASTQ-dump function (SRA-toolkit). Then, cellular barcode identification and unique molecular identifier (UMI) demultiplexing were performed. For droplet-based techniques such as 10X Genomics Chromium and Drop-seq, this procedure was performed by using UMI-tools (v1.1.2) [15], which identify and extract cell barcodes from the data. Specifically, first, cell barcodes were extracted and a putative whitelist was generated using the UMI-tools whitelist command. Note that the parameters of the “—bc-pattern” varied depending on the platform. For instance, the “—bc-pattern” option was set to “CCCCCCCCCCCCCCCCNNNNNNNNNN” for 10X 3′ v2 data and 10X 5′ data, while it was set to “CCCCCCCCCCCCCCCCNNNNNNNNNNNN “ for 10X 3′ v3 data. For Drop-seq data, the same command was used, except that the “—bc-pattern” option was set to “CCCCCCCCCCCCNNNNNNNN". Collapsing of the UMIs was performed using the umi_tools extract command, and the parameters of the “—bc-pattern” were also set depending on the platform, as described above. For techniques that are not based on droplet-based approaches such as Seq-Well, we developed an in-house Python script for cellular barcode identification and UMI demultiplexing.

The raw FASTQ data were quality trimmed using fastp (v0.23.1) [16] based on the read quality, complexity, and length. Low-quality reads were disregarded if their percentage of qualified bases was less than 40%, their complexity was less than 30, or their number of N bases was more than 5. Moreover, the polyA tail was trimmed for the sequence data, after which shorter reads with length less than 20 were removed.

The DVsc framework consists of two steps for sequencing read mapping. The cleaned reads were first aligned to the host reference genome database to subtract the host sequences using HISAT2 with relatively loose alignment parameters. The retained sequences were subsequently mapped to the viral reference database with relatively stringent aligning parameters by using HISAT2 (or other options of sequence aligners), and the viral reads were identified using SAMtools (v1.10) [17].

### Analysis process of the bulk RNA sequencing dataset

The datasets used were downloaded from the GEO database (https://www.ncbi.nlm.nih.gov/geo/), and the raw sequencing read files were split and extracted using FASTQ-dump. The FASTQ data were subsequently quality trimmed and mapped as described for the scRNA-seq dataset.

### Quality control filtering of the detected viral reads

As a part of the virus identification process in DVsc, virus segments with a sufficient number of mapped reads are filtered according to in-house quality control (QC) strategies, which are based on the sequencing mapping number, sequencing mapping quality, continuously mapped regions, and genome coverage. The first virus segment with 3 or more mapped reads was detected using SAMtools, and then, we filtered the virus using three features: the genome coverage, the number of qualified reads, and the length of continuously mapped regions. We analyzed these three features of expected and unexpected viruses in samples containing the given viruses, defined as positive viruses and negative viruses, respectively. To determine the most appropriate threshold for each feature, we calculated the value of each feature at different percentiles for all the negative viruses. This showed that at first, the growth of the feature was very slow as the percentile increased. When the percentile reached an inflection point, or ‘knee’, the feature suddenly increased sharply (Figure S1A). Hence, the values of the three features at this point were set as the threshold. Specifically, viruses with genome coverage less than 0.011554 or with qualified reads fewer than 10 were excluded from the analysis. Moreover, outliers were removed according to the sequence length. If the sequence length is not exceeding 75 bp, the length of the continuously mapped regions should not be less than 50 bp, and if the sequence length is exceeding 75 bp, the length of continuously mapped regions should not be less than 165.925 bp. As a result of the aforementioned filtering, false positives in the viral read detection were eliminated. For samples with more than one virus detected, the second filter was applied, which differed depending on whether the sample was analyzed by single-cell RNA-seq or by bulk RNA sequencing (RNA-seq). For single-cell RNA-seq, if the number of mapped reads was less than 50, the virus was excluded. For bulk RNA-seq, we analyzed two features: the number of each virus and the percentage of each virus in a sample, which was calculated as follows:

(1) $$\mathrm{Percent}\;\mathrm{of}\;\mathrm{virus}=N_{i}/N_{t}$$

where $N_{i}$ is the number of the given virus and $N_{t}$ is the total number of detected viruses. These two features were calculated for infected samples and control samples, defined as positive viruses and negative viruses, respectively. Boxplots showed that for both features, the number and percentage of positive viruses were much greater than those of negative viruses (Figure S1B). Therefore, we selected an integer value close to the minimum number of positive viruses as the threshold, which means that the virus is deleted if its $N_{i}$ is less than 2981 ($e^{8}$) and its percentage is less than 0.6 (60%).

A list of viruses was generated from the samples that passed the filtrations, and this list can then be subjected to further investigation. For the scRNA-seq dataset, the filtered viral reads were demultiplexed based on the UMIs and unique cell barcodes by using an in-house Python script and assigned to unique viral transcripts and infected cells. To correct potential bias from different cells, we normalized the number of virus transcripts ($N_{v}$) in each cell based on the total number of transcripts ($N_{T}$) in each cell, which was calculated as follows:

(2) $$N_{nor}=N_{v}\times 1000000/N_{T}\;$$

### Transcriptome assembly

The majority of complete virus transcriptomes are not available. Despite the constant discoveries of novel transcripts, the few published transcripts remain poorly annotated. Therefore, for all the viruses in the final list, the complete set of transcripts was reconstructed using *de novo* transcriptome assembly via StringTie [18].

### Performance evaluation

To evaluate the performance of our method compared with that of other pipelines that can also detect virus-transcribed mRNAs, we conducted a benchmark experiment using Viral-Track [13], Venus [19], and our proposed DVsc. Based on the publicly available scRNA-seq and bulk RNA-seq datasets that we collected, we divided all the samples into positive sample sets (infected samples) and negative sample sets (uninfected or mock samples). Since Venus can be used for only scRNA-seq datasets generated from the 10X Genomics platform, we included only single-cell sequencing data derived from the 10X Genomics platform. For scRNA-seq, we obtained a total of 55 samples, with 38 positive samples and 17 negative samples. For bulk RNA-seq, we obtained a total of 71 samples, with 39 positive samples and 32 negative samples. Using the DVsc, Viral-Track, and Venus methods, we calculated the positive and negative numbers based on the predicted results of each method. The predicted results of each method were then compared with the true labels of the samples. By comparing the predicted results with the ground truth, we calculated the accuracy of each method on the positive, negative, and overall samples.

### Reference file downloads

The Human RefSeq hg38 reference file was downloaded from http://ftp.ensembl.org/pub/release-105/fasta/homo_sapiens/dna/Homo_sapiens.GRCh38.dna.toplevel.fa.gz. The mitochondrial genome of human was downloaded from https://www.ncbi.nlm.nih.gov/nuccore/251831106. The viruSITE reference sequences were downloaded from http://www.virusite.org/index.php?nav=download. The virus RefSeq data from NCBI were downloaded from https://www.ncbi.nlm.nih.gov/genomes/GenomesGroup.cgi?taxid=10239.

### Implementation of the web server

To provide an online data analysis platform, a web server was constructed using Flask, a Python web framework. The web server acts as the backend for the website, allowing users to interact with the data analysis functionality. Additionally, the website provides the option to download and access the local version, which includes the source code and the needed databases. To facilitate the use of the local version, we implemented automatic installation of the needed software dependencies and streamlined the analysis process to be executed in a single script.

### Availability of data or materials

All the datasets obtained and analyzed in this study are available from public databases (Table S1).

## Results

### Pipeline design of DVsc

**Figure 1:**
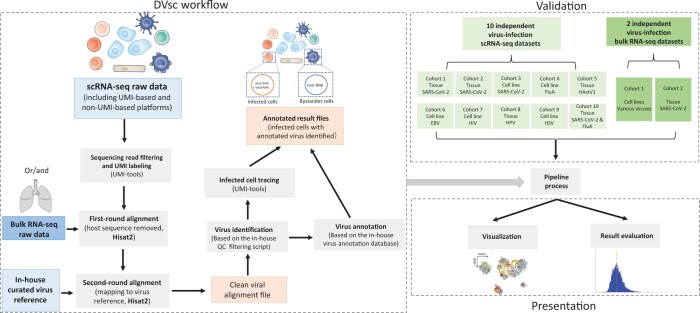
Illustration of the study workflow Flowchart of the data collection, method implementation, and validation steps in this work. RNA-seq, RNA sequencing; scRNA-seq, single-cell RNA sequencing; UMI, unique molecular identifier; QC, quality control.

The workflow of DVsc is shown in **Figure 1**. DVsc accepts single-cell RNA-seq files in FASTQ format. For the scRNA-seq reads, demultiplexing was needed before mapping. The raw FASTQ data were subsequently quality trimmed based on the quality, length, and complexity of the sequencing reads (**Table 1**). The read mapping module in the DVsc framework consists of two steps. In the first step, the clean sequencing reads are mapped to the host reference genome database, which includes the combined human reference genome and mitochondrial genome, to remove the host RNA-seq reads. In the second step, the filtered sequencing reads are mapped to the high-quality curated viral genomes. Since viral reads are highly repetitive and can produce significant sequencing artifacts [13], the viruses identified in DVsc with a sufficient number of mapped reads were then filtered based on in-house quality QC strategies: the sequencing mapping number, sequencing mapping quality, continuously mapped regions, and genome coverage. After detecting the first virus segment with at least 5 mapped reads, false positives in the viral read detection were eliminated using in-house scripts (see details in Method). For samples with more than one virus detected, a second run of the filtering module was performed. Ultimately, the filtered viral reads were demultiplexed based on the UMIs and unique cell barcodes and were assigned to the corresponding viral transcripts and infected cells.

**Table 1:** Overview of the datasets used in this study

Dataset group	Dataset name	Source	Virus	Platform	No. of infected samples	Tissue/cell line	Pubmed ID
scRNA-seq for SARS-CoV-2	COVID19_1	Colon organoids	SARS-CoV-2	10X 3′ v3	2	Tissue	33904651
	COVID19_2	Ileum organoids	SARS-CoV-2	10X 3′ v3	2	Tissue	33904651
	COVID19_3	BALF	SARS-CoV-2	10X 5′	9	Tissue	32398875
	COVID19_4	Human bronchial epithelial cells	SARS-CoV-2	10X 3′ v3	3	Cell line	33730024
scRNA-seq for other viruses	H1N1	A549 cells	H1N1	10X 3′ v2	2	Cell line	32614923
	H3N2	A549 cells	H3N2	10X 3′ v3	2	Cell line	32614923
	HAstV1	Ileum organoids	HAstV1	10X 3′ v2 and v3	8	Tissue	34309190
	EBV	B cells	EBV	10X 3′ v2	3	Cell line	33501914
	HIV	CD4^+^ T cells	HIV	10X Genomics	2	Cell line	30282021
	HPV	HNSCC primary tumors	HPV	10X 3′ v2	8	Tissue	31924475
scRNA-seq for other platforms	DropSeq	NHDF primary human fibroblasts	HSV	Drop-seq	14	Cell line	31653857
	SeqWell1	Saline nasal wash cells	SARS-CoV-2	Seq-Well	32	Tissue	–
	SeqWell2	Saline nasal wash cells	H1N1	Seq-Well	56	Tissue	–
Bulk RNA-seq	Bulkcell1	A549 cells	SARS-CoV-2	–	6	Cell line	32416070
	Bulkcell2	A549-ACE2 cells	SARS-CoV-2	–	9	Cell line	32416070
	Bulkcell3	NHBE cells	SARS-CoV-2	–	3	Cell line	32416070
	Bulkcell4	Calu-3 cells	SARS-CoV-2	–	3	Cell line	32416070
	Bulkcell5	A549 cells	H1N1	–	2	Cell line	32416070
	Bulkcell6	NHBE cells	H1N1	–	4	Cell line	32416070
	Bulkcell7	NHBE cells	H1N1 lacks NS1	–	4	Cell line	32416070
	Bulkcell8	A549 cells	HPIV3	–	3	Cell line	32416070
	Bulkcell9	A549 cells	RSV	–	5	Cell line	32416070
	Bulk tissue	BALF	SARS-CoV-2	–	4	Tissue	32228226

*Note*: scRNA-seq, single-cell RNA sequencing; COVID, coronavirus disease; SARS-CoV-2, severe acute respiratory syndrome coronavirus 2; BALF, bronchoalveolar lavage fluid; HAstV1, human astrovirus 1; EBV, Epstein–Barr virus; HIV, human immunodeficiency virus; HPV, human papillomavirus; HNSCC, head and neck squamous cell carcinoma; HSV, herpes simplex virus; HPIV3, parainfluenza virus type 3; RSV, respiratory syncytial virus.

### Characterization of viral infections in single-cell transcriptomics data

**Figure 2:**
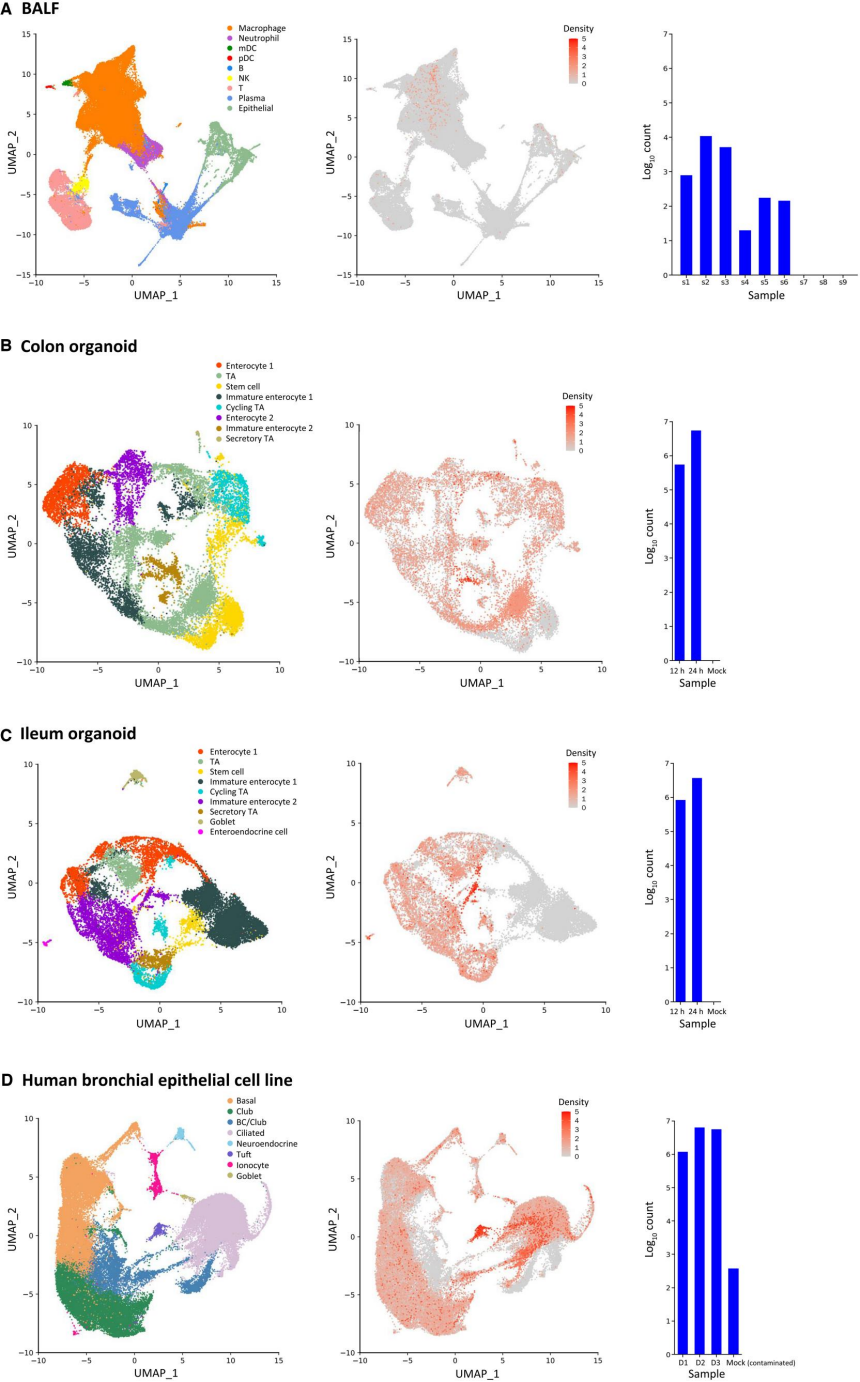
Discovery of SARS-CoV-2 infection in human samples and cell lines BALF (**A**), colon organoid (**B**), ileum organoid (**C**), and human bronchial epithelial cell line (**D**) samples infected with SARS-CoV-2. Left: 2D visualization of cells annotated by distinct clusters of cell phenotypes from SARS-CoV-2-infected clinical samples and cell lines. Middle: the density of viral reads across all the cells projected on the 2D map from corresponding samples. Right: a bar plot showing the abundance of viral infection across all corresponding samples. SARS-CoV-2, severe acute respiratory syndrome coronavirus 2; BALF, bronchoalveolar lavage fluid; mDC, myeloid dendritic cell; pDC, plasmacytoid dendritic cell; NK, natural killer cell; TA, transient amplifying cell; BC, basal cell; D, day.

We further examined the applicability of the DVsc workflow based on real-world datasets for detecting viral reads in human clinical samples infected with various types of viruses (Table 1). Severe acute respiratory syndrome coronavirus 2 (SARS-CoV-2), which is the causative agent of coronavirus disease 2019 (COVID-19), has infected more than 2.3 million people and caused worldwide social and economic disruption [20]. We collected the available scRNA-seq clinical samples with raw sequencing data from patients infected by SARS-CoV-2 and then performed DVsc analysis to evaluate the practicability of our pipeline and gain insights into the infection course of SARS-CoV-2. We collected and analyzed the scRNA-seq datasets of bronchoalveolar lavage fluid (BALF) samples, colon and ileum organoids, and human bronchial epithelial cell lines infected with SARS-CoV-2. The unified expression profiles of all the cells profiled in the scRNA-seq datasets were compiled, and the cell populations were subsequently identified and visualized using the uniform manifold approximation and projection (UMAP) algorithm provided in the “scRNA-seq preprocessing” module. To identify infected and bystander populations, the filtered viral data were then overlaid on the host transcriptome. As shown in **Figure 2**, DVsc successfully detected valid sequencing reads of SARS-CoV-2 from patients with severe COVID-19 (Figure 2A), from colon and ileum organoid samples (Figure 2B), and from all infected human bronchial epithelial cell lines (Figure 2C). In many cases of infectious disorders, the infecting virus is accompanied by coinfection with unknown viruses. By analyzing the data from one of the patients with severe COVID-19 using DVsc, another virus, human metapneumovirus (hMPV), was identified. It was detected in more than one million valid viral reads in the sample (Figure S2). Therefore, these results strongly indicate that DVsc is an optimal method for systematically profiling the source of infection or coinfection in human clinical samples.

**Figure 3:**
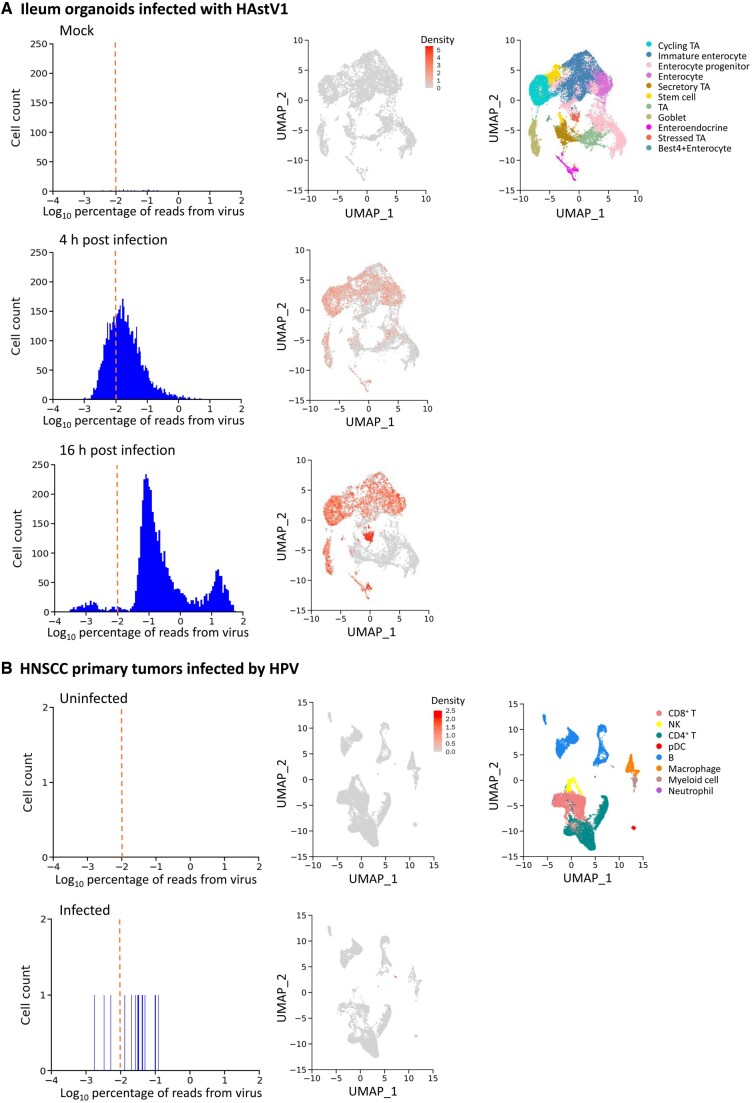
Discovery of virus infection in human samples based on scRNA-seq transcriptomic data **A**. Analysis of ileum organoids infected with HAstV1. Left: density plots of the detected viral reads across cell numbers from mock-infected, 4-h infected, and 16-h infected samples. Middle: enrichment of viral reads projected on the 2D map from the corresponding sample. Right: 2D visualization of cells annotated by distinct clusters of cell phenotypes. **B**. Analysis of HNSCC primary tumors infected by HPV. Left: density plots of the detected viral reads across cell numbers from uninfected and infected samples. Middle: enrichment of viral reads projected on the 2D map from the corresponding sample. Right: 2D visualization of cells annotated by distinct clusters of cell phenotypes. HAstV1, human astrovirus 1; HNSCC, head and neck squamous cell carcinoma; HPV, human papillomavirus.

**Figure 4:**
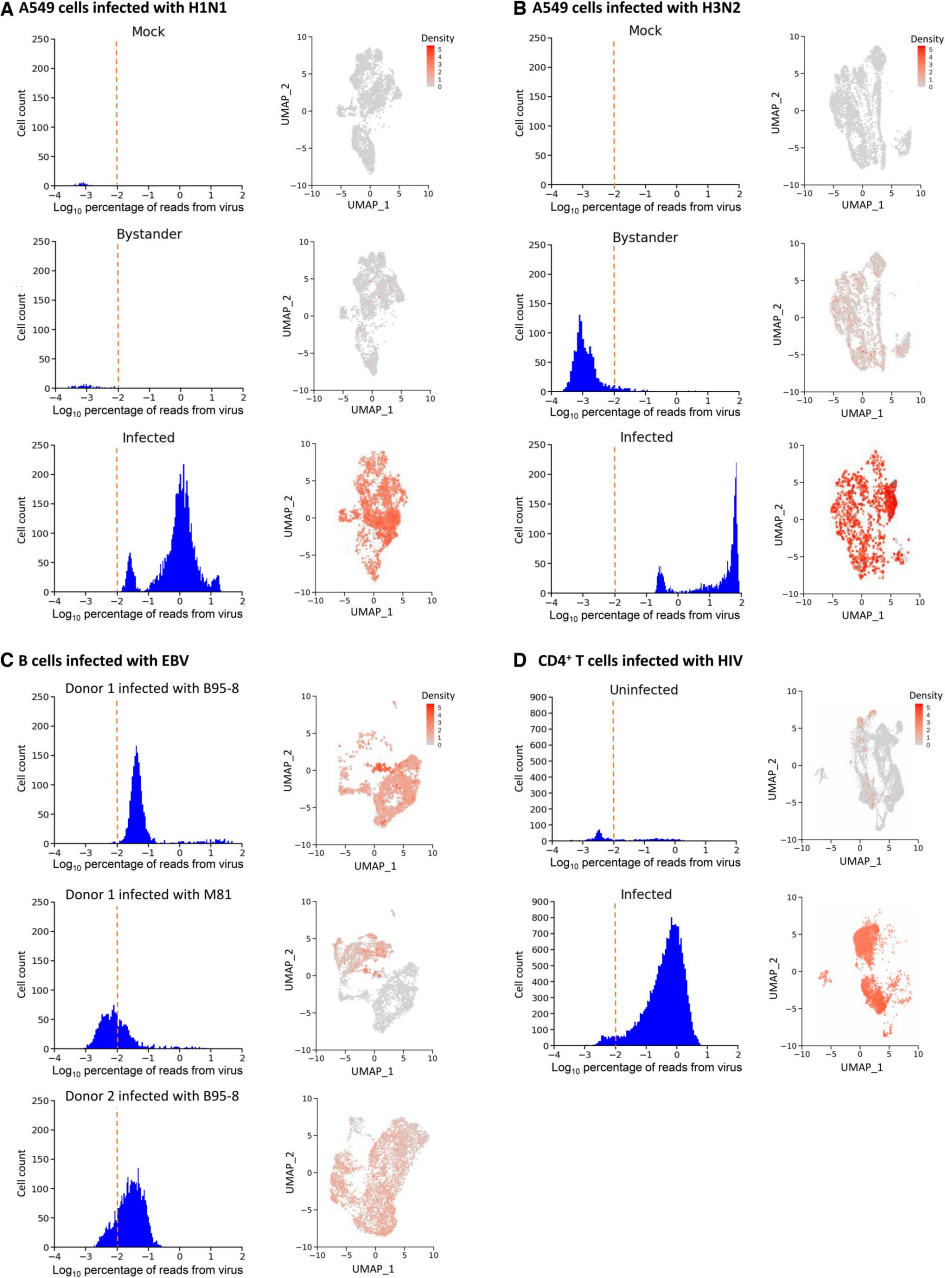
Discovery of virus infection in cell line samples based on scRNA-seq transcriptomic data **A**. Analysis of A549 cells infected with H1N1. Left: density plots of the detected viral reads across cell numbers from mock-infected, bystander, and infected cells. Right: enrichment of viral reads projected on the 2D map from the corresponding sample. **B**. Analysis of A549 cells infected with H3N2. Left: density plots of the detected viral reads across cell numbers from mock-infected, bystander, and infected cells. Right: enrichment of viral reads projected on the 2D map from the corresponding sample. **C**. Analysis of B cells infected with EBV. Left: density plots of the detected viral reads across cell numbers from donor 1 infected with the EBV strain B95-8, donor 1 infected with the EBV strain M81, and donor 2 infected with the EBV strain B95-8. Right: enrichment of viral reads projected on the 2D map from the corresponding sample. **D**. Analysis of CD4^+^ T cells infected with HIV. Left: density plots of the detected viral reads across cell numbers from uninfected and infected cells. Right: enrichment of viral reads projected on the 2D map from the corresponding sample. EBV, Epstein–Barr virus; HIV, human immunodeficiency virus.

Next, we benchmarked DVsc on a greater number of public datasets using the 10X Genomics platform to evaluate the sensitivity of our pipeline over a broader range. These datasets comprise a large number of publicly accessible studies of various sample sources with different virus infections, which cover a wide range of cell types (including B, T, and tumor cells), as well as a broad range of viruses, namely, human astrovirus 1 (HAstV1; **Figure 3**A), human papillomavirus (HPV; Figure 3B), influenza A viruses (IAVs; including H1N1 and H3N2; **Figure 4**A and B), Epstein‒Barr virus (EBV; Figure 4C), and human immunodeficiency virus (HIV; Figure 4D). In summary, DVsc detected all infected samples via accurate host−virus infection mapping. These results suggest that our analysis framework, DVsc, is a highly sensitive and reliable tool for identifying viral reads and characterizing virus-infected cells based on scRNA-seq data.

### Detection of viral infection across different scRNA-seq platforms

**Figure 5:**
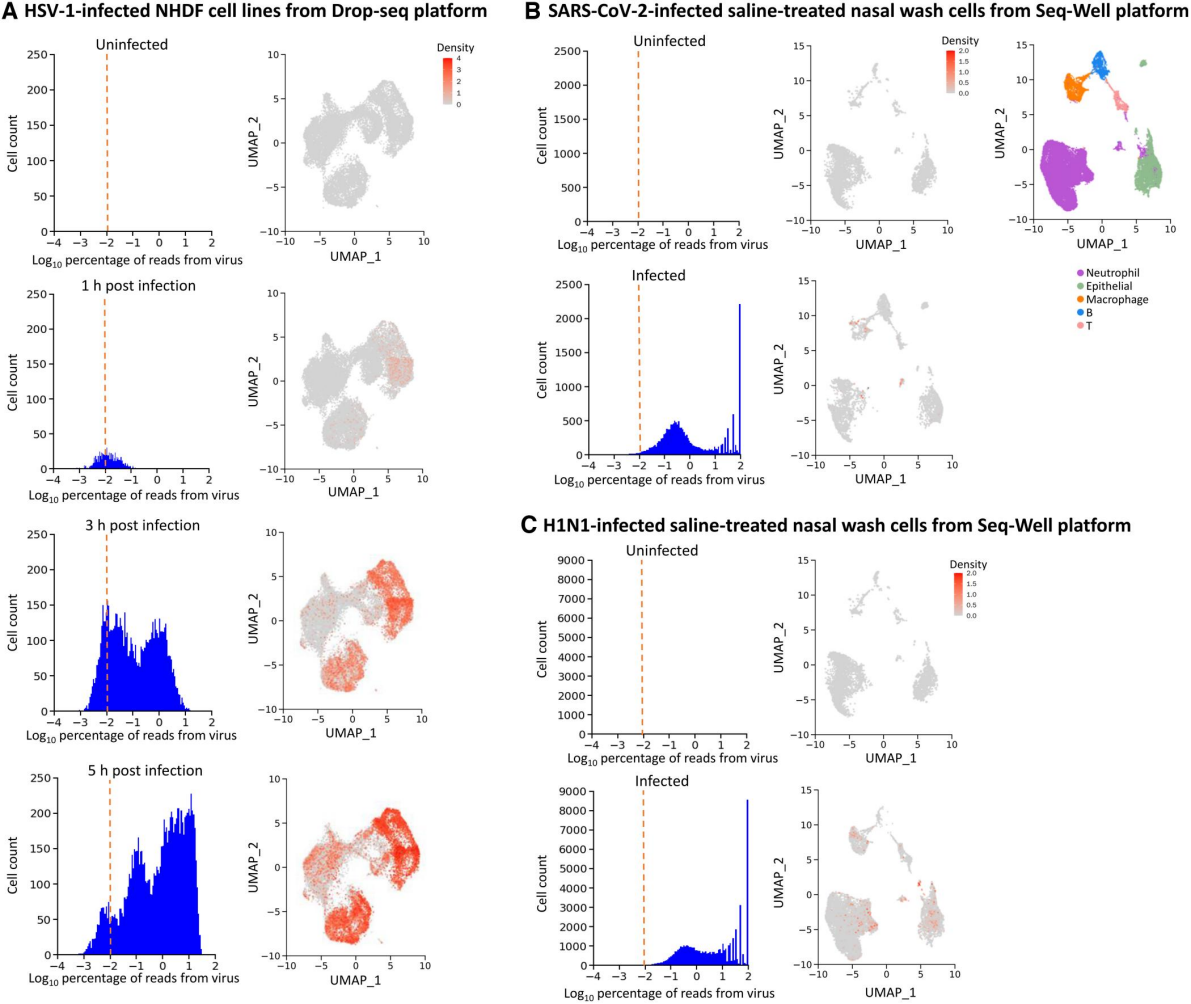
Performance evaluation of viral read discovery across different scRNA-seq platforms **A**. Analysis of HSV-1-infected NHDF cell lines from Drop-seq platforms. Left: density plots of the detected viral reads across cell numbers from uninfected, 1-h infected, 3-h infected, and 5-h infected cells. Right: enrichment of viral reads projected on the 2D map from the corresponding sample. **B**. Analysis of SARS-CoV-2-infected saline-treated nasal wash cells from Seq-Well platforms. Left: density plots of the viral reads detected across cell numbers from uninfected and infected samples. Middle: enrichment of viral reads projected on the 2D map from the corresponding sample. Right: 2D visualization of cells annotated by distinct clusters of cell phenotypes. **C**. Analysis of H1N1-infected saline-treated nasal wash cells from Seq-Well platforms. Left: density plots of the detected viral reads across cell numbers from uninfected and infected samples. Right: enrichment of viral reads projected on the 2D map from the corresponding sample. HSV-1, herpes simplex virus-1.

To construct high-throughput sequencing libraries, scRNA-seq approaches require the isolation and lysis of single cells, the conversion of RNA into cDNA, and the amplification of cDNA. Because diverse scRNA-seq strategies have inherent strengths and weaknesses, distinct protocols based on different scRNA-seq platforms could result in substantial technical variation. Thus, we employed our DVsc pipeline for two other prominent scRNA-seq methods, Drop-seq and Seq-Well. **Figure 5**A shows the results of the scRNA-seq experiment performed on human primary fibroblasts infected with herpes simplex virus 1 (HSV-1) using the Drop-seq platform at different time points after infection. By analyzing the raw sequencing data, we identified infected cells with a gradual increase in the number of viral reads (Figure 5A). Similarly, another scRNA-seq dataset of nasal wash cells that was collected from adults infected with SARS-CoV-2 or IAV (H1N1) and from healthy donors was analyzed using the Seq-Well platform, and our DVsc pipeline also performed successfully in detecting the viral reads and the infected cell type (Figure 5B and C). Together, these extensive validations demonstrate that DVsc is a sensitive and accurate framework for detecting and identifying viral infection across diverse scRNA-seq platforms, across different tissues, and across varying viral types and loads.

### Application of DVsc to bulk RNA-seq analyses

**Figure 6:**
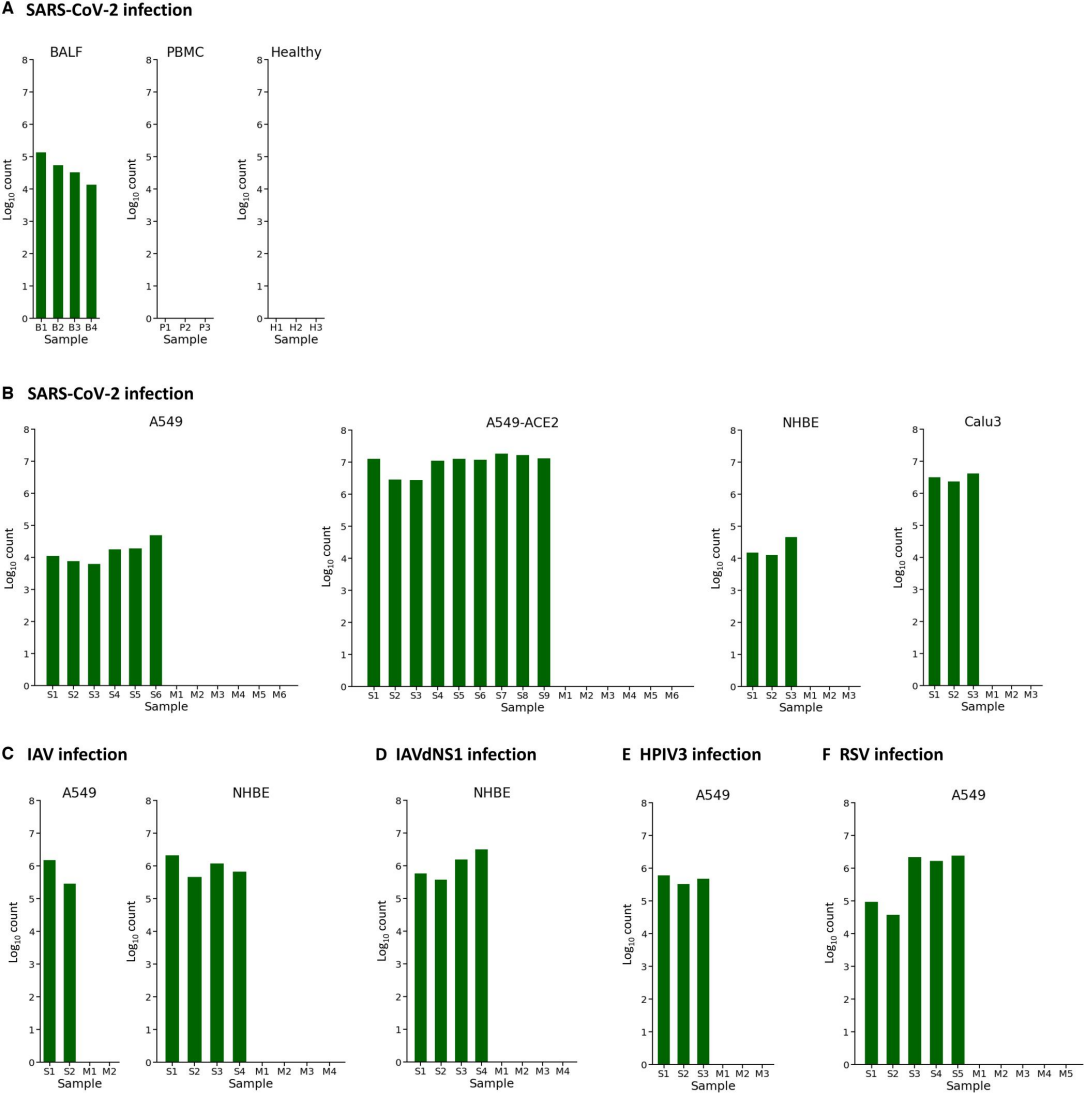
Performance evaluation of viral read discovery based on bulk RNA-seq data Bar plots revealed the abundance of the detected viral reads across different samples based on the bulk transcriptomic data. **A**. Viral reads were determined in BALF and PBMC samples infected with SARS-CoV-2 and in healthy control samples. **B**. Vrial reads were detected in A549, A549-ACE2, NHBE, and Calu3 cells infected with SARS-CoV-2. **C**. Vrial reads were detected in A549 and NHBE cells infected with IAV. **D**. Vrial reads were detected in NHBE cells infected with IAVdNS1. **E**. Vrial reads were detected in A549 cells infected with HPIV3. **F**. Viral reads were detected in A549 cells infected with RSV. PBMC, peripheral blood mononuclear cell; NHBE, normal human bronchial lung epithelial cell; IAV, influenza A virus; IAVdNS1, IAV with a null interferon antagonist NS1 mutant; HPIV3, parainfluenza virus type 3; RSV, respiratory syncytial virus.

To further assess the applicability of DVsc to bulk RNA-seq analyses, we applied our DVsc pipeline to various RNA-seq datasets of multiple tissue samples and cell lines infected by different types of viruses. RNA isolated from the BALF and peripheral blood mononuclear cell (PBMC) specimens of COVID-19 patients and healthy donors was investigated via transcriptome sequencing. The DVsc pipeline was applied to the collected dataset to analyze the raw sequencing files. Sufficient numbers of SARS-CoV-2 viral reads were detected by DVsc in the BALF samples of COVID-19 patients, but no valid viral reads were detected in the PBMC samples of COVID-19 patients or in any healthy donor samples (**Figure 6**A). Similarly, as shown in Figure 6B–F, bulk RNA-seq data of independent biological replicates of the primary normal human bronchial lung epithelial cells (NHBE), transformed lung alveolar (A549) cells transduced with/without a vector expressing human angiotensin-converting enzyme 2 (ACE2), and transformed lung-derived Calu-3 (Calu3) cells that were mock-treated or infected with SARS-CoV-2, IAV [A/Puerto Rico/8/1934 (H1N1)], IAV that lacked the NS1 protein (IAVdNS1), RSV (A2 strain), or human parainfluenza virus type 3 (HPIV3) were analyzed by DVsc. The results showed that the DVsc pipeline could detect all the infected samples with precise viral read counts. In summary, DVsc is an effective framework for the quantitative analysis of viral infection from both scRNA-seq and bulk RNA-seq data. Importantly, DVsc can be applied to human clinical samples to obtain valuable insights into the biology of host−virus interactions.

### Performance evaluation based on a benchmarking study

A benchmarking study was conducted to evaluate the viral detection performance of three recently released methods, namely, Venus, Viral-Track, and our proposed pipeline DVsc. The benchmarking was performed on both scRNA-seq and bulk RNA-seq datasets, and we assessed the accuracy of each method for detecting virus-transcribed mRNAs. Table S2 provides the performance results of the three methods. For scRNA-seq data, DVsc achieved an accuracy of 79% for positive samples and 82% for negative samples, while Viral-Track achieved an accuracy of 71% and 82%, respectively, and Venus achieved an accuracy of 84% and 29%, respectively. Overall, on the combined scRNA-seq dataset, DVsc had an accuracy of 80%, Viral-Track had an accuracy of 75%, and Venus had an accuracy of 67%. For bulk RNA-seq data, all three methods achieved 100% accuracy on positive samples; however, there was a significant difference between the accuracy for negative samples: DVsc achieved 100% accuracy, Viral-Track achieved 53% accuracy, and Venus achieved 19% accuracy on negative samples. These results indicate that DVsc consistently performs well on both scRNA-seq and bulk RNA-seq datasets. However, while Venus showed promising accuracy on positive samples, especially in the scRNA-seq dataset, it struggled to perform well on negative samples.

## Discussion

Viruses are obligate intracellular pathogens that rely on the host cell machinery to survive; viruses attach to and enter target host cells through interactions between viral attachment proteins and receptors on the host cell surface [21,22]. For example, SARS-CoV-2 utilizes its spike glycoprotein to recognize and bind to host ACE2 to enter host cells [23,24]. SARS-CoV-2 infects multiple tissues that express ACE2, such as the lung, oral cavity, vasculature, heart, kidney, gastrointestinal tract, pancreas, and brain [25]. Once internalization is complete, viruses can utilize the host and its factors to replicate their genetic material, assemble new viral particles, and release them to infect new host cells. Some viruses can integrate their genomes into host chromosomes, become dormant if necessary, and replicate under certain circumstances [26]. Therefore, flexible methods are needed for the detection of SARS-CoV-2 RNA. In addition to entry receptors, interactions between viruses and host proteins occur constantly during virus intracellular life cycles, such as interactions with vesicular trafficking factors, which are needed for virus RNA synthesis, virus assembly, and viral mRNA translation [27]. These interactions are closely associated with the host immune response and pathological changes. However, it has been extremely challenging to analyze the virus status in host tissues, particularly in terms of the endogenous expression of host genes. Furthermore, because viral infection varies according to the cell population, it is unknown which cells are infected, how many virus species are present, and what conditions viruses and host cells are in. scRNA-seq technology is an effective method for elucidating viral pathogenicity and interactions between viruses and host cells. For example, specific cell subsets targeted by SARS-CoV-2 and the factors that regulate ACE2 expression were detected in host tissues by scRNA-seq [28]. Single-cell V(D)J sequencing also plays an important role in identifying SARS-CoV-2-neutralizing antibodies [29]. Moreover, some single-cell atlases are already fairly complete for the exploration of host−virus interactions.

The lack of computation time evaluation based on different computational platforms and resources is a limitation of this work. The analyses in this study were all performed based on the same device with AMD processor 7742 and 1024 GB of RAM (DDR4), as well as the CentoS7 system. We believe that the analysis indicates that the runtime depends not only on the size of the dataset (the number of cells and the number of sequencing reads) but also on the computing environment. Therefore, in our future work, we will perform a systematic running time evaluation across different computing resources based on scRNA-seq datasets with various complexities. The lack of a polyA tail at the end of viral RNA molecules can significantly lower the capture rate of viruses via current scRNA-seq techniques, which may increase the difficulty of distinguishing between infected and bystander cells or accurately identifying infected cells via DVsc. As scRNA-seq technology develops, researchers should take this limitation into account and strive to improve the representation and categorization of molecular traits that aid or hinder the detection of viruses. On the other hand, contamination is a plausible source, whether in scRNA-seq or bulk RNA-seq experiments. Although strict filtering conditions were set, false positives were still unavoidable. In our analysis, there were 49 virus-negative samples (including mock samples and uninfected samples), of which 4 samples were confirmed to contain the target virus at a false-positive rate of 8%. It is worth noting that the virus content in these false-positive samples was much lower than that in the true-positive samples of the same group.

According to our findings, bulk RNA-seq data from external sources are a reference for detecting viral infections. By comparing the expression profiles of single cells with bulk RNA-seq data, we were able to detect viral infection and assess the degree of viral involvement [30,31]. Furthermore, leveraging single-cell transcriptomics data from similar tissues as a reference holds significant promise, given the extensive efforts of cell atlas consortia in generating massive amounts of single-cell transcriptomics data [32,33]. This strategy has been successfully applied to challenging spatial transcriptomics analyses [34,35]. By utilizing single-cell data from similar tissues as a reference, we can gain insights into cell type-specific responses to viral infection and further enhance our understanding of the impact of viral infection on specific cell types within tissues or organs. Moreover, future investigations should involve the analysis of spatial transcriptomics data.

We attempted to develop a technique for simultaneously monitoring multiple viral transcriptomes within a single cell. This technique was evaluated using data collected from various scRNA-seq platforms. These data included infections caused by different types of viruses, each with distinct RNA characteristics, and originated from a variety of tissues and cell types. We showed that DVsc could quickly provide crucial details on the infection status of clinical samples, identify infected cells, investigate virally induced transcriptional modifications, and detect instances of coinfection. Our results demonstrate that DVsc is a robust and efficient computational framework capable of detecting viral RNA in any scRNA-seq dataset without the need for experimental modifications or prior knowledge of the infecting agent. With the use of this technique, we can uncover cells harboring activated viruses and the composition of multiple viruses in a cell and determine how the expression differs in infected and uninfected cells. By serving as a trigger or moderator of illness development, viruses contribute to the development of several diseases, and this study suggests viable treatment options by targeting viruses.

## Code availability

DVsc is available at http://62.234.32.33:5000/DVsc, and all the codes for this study are available at https://ngdc.cncb.ac.cn/biocode/tools/BT007367.

## CRediT author statement

**Fei Leng:** Conceptualization, Methodology, Software, Formal analysis, Investigation, Data curation, Writing – original draft, Writing – review & editing. **Song Mei:** Methodology, Writing – original draft. **Xiaolin Zhou:** Methodology, Validation. **Xuanshi Liu:** Methodology, Validation. **Yefeng Yuan:** Methodology, Validation. **Wenjian Xu:** Methodology, Validation. **Chongyi Hao:** Methodology, Validation. **Ruolan Guo:** Methodology, Validation. **Chanjuan Hao:** Conceptualization, Methodology, Validation, Resources, Writing – original draft, Writing – review & editing, Supervision, Project administration. **Wei Li:** Conceptualization, Methodology, Validation, Resources, Writing – original draft, Writing – review & editing, Supervision, Project administration. **Peng Zhang:** Conceptualization, Methodology, Validation, Resources, Writing – original draft, Writing – review & editing, Supervision, Project administration. All authors have read and approved the final manuscript.

## Supplementary material

Supplementary material is available at *Genomics, Proteomics & Bioinformatics* online (https://doi.org/10.1093/gpbjnl/qzad007).

## Competing interests

The authors have declared no competing interests.

## Supplementary Material

qzad007_Supplementary_Data
