# Supplementary material for: DVsc: An Automated Framework for Efficiently Detecting Viral Infection from Single-cell Transcriptomics Data
Source: Genomics Proteomics Bioinformatics. 2023 Dec 19;22(2):qzad007. doi: 10.1093/gpbjnl/qzad007 (PMC12016032; doi:10.1093/gpbjnl/qzad007)
Supplement: qzad007_Supplementary_Data [file qzad007_supplementary_data.zip › Supplementary material captions.docx]

**Supplementary material**

**Figure S1 Estimated feature distribution for the filtering step**

**A.** Normalized values of features (genome coverage of the virus, number of qualified sequencing reads and length of continuously mapped regions) at different percentiles (top). Statistics of features of negative and positive viruses (bottom). **B.** Statistics of features based on control samples (with no virus infection) and infected samples.

**Figure S2 Discovery of virus infection in coinfected human samples**

A density plot and UMAP visualization of the detected viral reads based on single-cell transcriptomics data of coinfected human samples. UMAP, uniform manifold approximation, and projection.

**Table S1 Accession codes of the datasets used in this work**

**Table S2**  **Benchmarking results based on different methods**
