# Supplementary material for: DVsc: An Automated Framework for Efficiently Detecting Viral Infection from Single-cell Transcriptomics Data
Source: Genomics Proteomics Bioinformatics. 2023 Dec 19;22(2):qzad007. doi: 10.1093/gpbjnl/qzad007 (PMC12016032; doi:10.1093/gpbjnl/qzad007)
Supplement: qzad007_Supplementary_Data [file qzad007_supplementary_data.zip › Table S1.docx]

**Table S1 Accession codes of the datasets used in this work**

| **Datasets name** | **Accession code** |
| --- | --- |
| COVID19_1 | GEO: GSE156760 |
| COVID19_2 | GEO: GSE156760 |
| COVID19_3 | GEO: GSE145926 |
| COVID19_4 | GEO: GSE166766 |
| H1N1 | GEO: GSE143167 |
| H3N2 | GEO: GSE143167 |
| HAstV1 | GEO: GSE171620 |
| EBV | GEO: GSE158275 |
| HIV | SAMN08685499, SAMN08685500, SAMN08685501, SAMN08685502 |
| HPV | GEO: GSE139324 |
| Dropseq | GEO: GSE123782 |
| SeqWell1 | GEO: GSE176269 |
| SeqWell2 | GEO: GSE176269 |
| Bulkcell1 | GEO: GSE147507 |
| Bulkcell2 | GEO: GSE147507 |
| Bulkcell3 | GEO: GSE147507 |
| Bulkcell4 | GEO: GSE147507 |
| Bulkcell5 | GEO: GSE147507 |
| Bulkcell6 | GEO: GSE147507 |
| Bulkcell7 | GEO: GSE147507 |
| Bulkcell8 | GEO: GSE147507 |
| Bulkcell9 | GEO: GSE147507 |
| Bulk tissue | GSA: CRA002390 |

*Note*: EBV, Epstein‒Barr virus; HIV, human immunodeficiency virus; HPV, human papillomavirus; COVID, coronavirus disease; GEO, Gene Expression Omnibus; GSA, Genome Sequence Archive.
