# Supplementary material for: DVsc: An Automated Framework for Efficiently Detecting Viral Infection from Single-cell Transcriptomics Data
Source: Genomics Proteomics Bioinformatics. 2023 Dec 19;22(2):qzad007. doi: 10.1093/gpbjnl/qzad007 (PMC12016032; doi:10.1093/gpbjnl/qzad007)
Supplement: qzad007_Supplementary_Data [file qzad007_supplementary_data.zip › Table S2.docx]

**Table S2 Benchmarking results based on different methods**

| **Group** | **Samples type for benchmark** | **Samples number for benchmark** | **Accurate predicted number (percentage) by methods** | | |
| --- | --- | --- | --- | --- | --- |
|  |  |  | **DVsc** | **viral_track** | **Venus** |
| scRNA-seq | Positive samples | 38 | 30 (0.79) | 27 (0.71) | 32 (0.84) |
|  | Negative samples | 17 | 14 (0.82) | 14 (0.82) | 5 (0.29) |
|  | Total samples | 55 | 44 (0.8) | 41 (0.75) | 37 (0.67) |
| Bulk RNA-seq | Positive samples | 39 | 39 (1) | 39 (1) | 39 (1) |
|  | Negative samples | 32 | 32 (1) | 17 (0.53) | 6 (0.19) |
|  | Total samples | 71 | 71 (1) | 56 (0.79) | 45 (0.63) |
