# Supplementary figures and images for: DVsc: An Automated Framework for Efficiently Detecting Viral Infection from Single-cell Transcriptomics Data
Source: Genomics Proteomics Bioinformatics. 2023 Dec 19;22(2):qzad007. doi: 10.1093/gpbjnl/qzad007 (PMC12016032; doi:10.1093/gpbjnl/qzad007)

## BALF infected by SARS-CoV-2 and HMPV

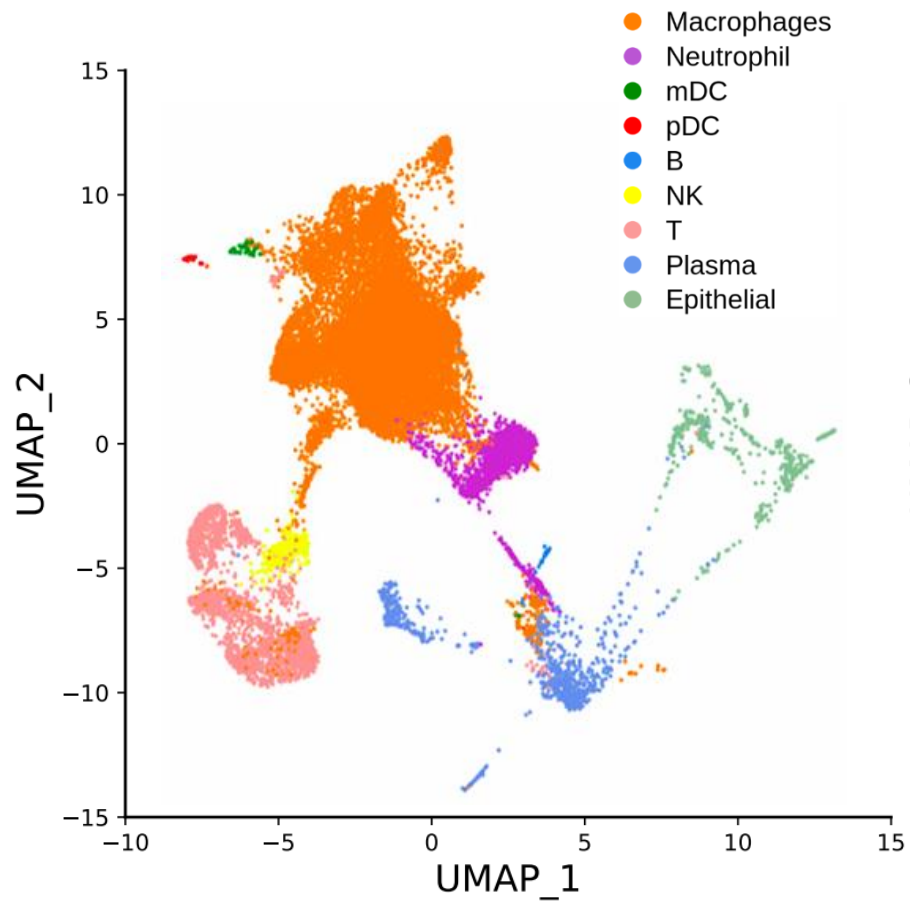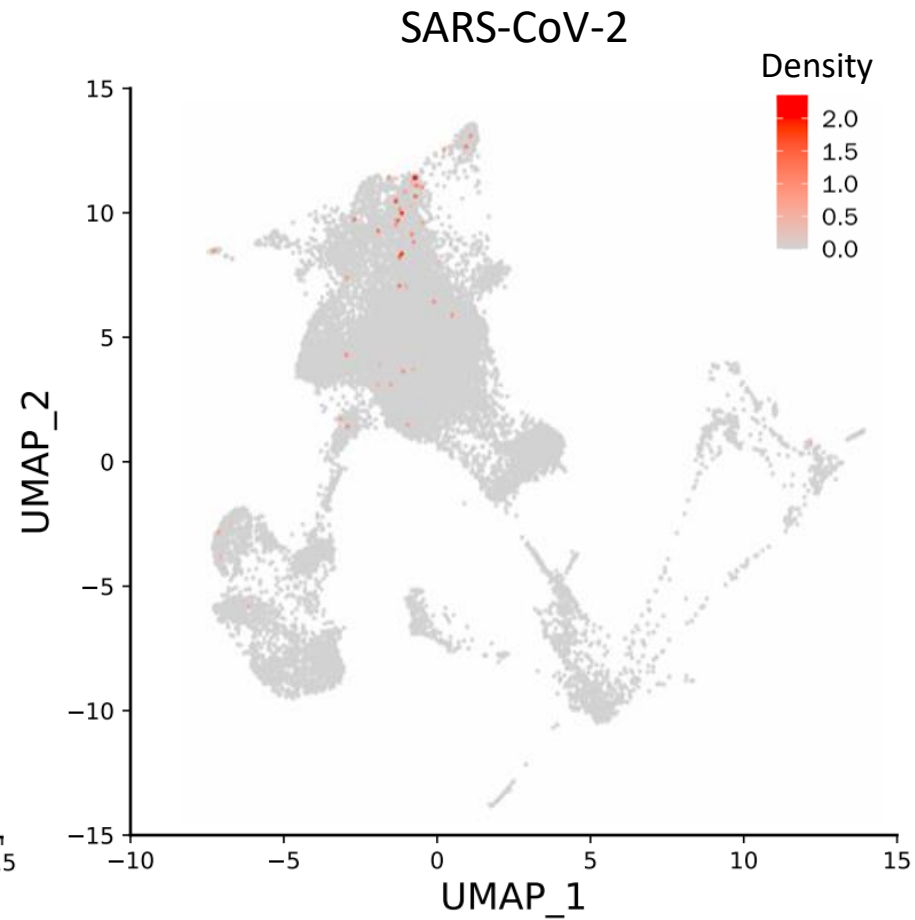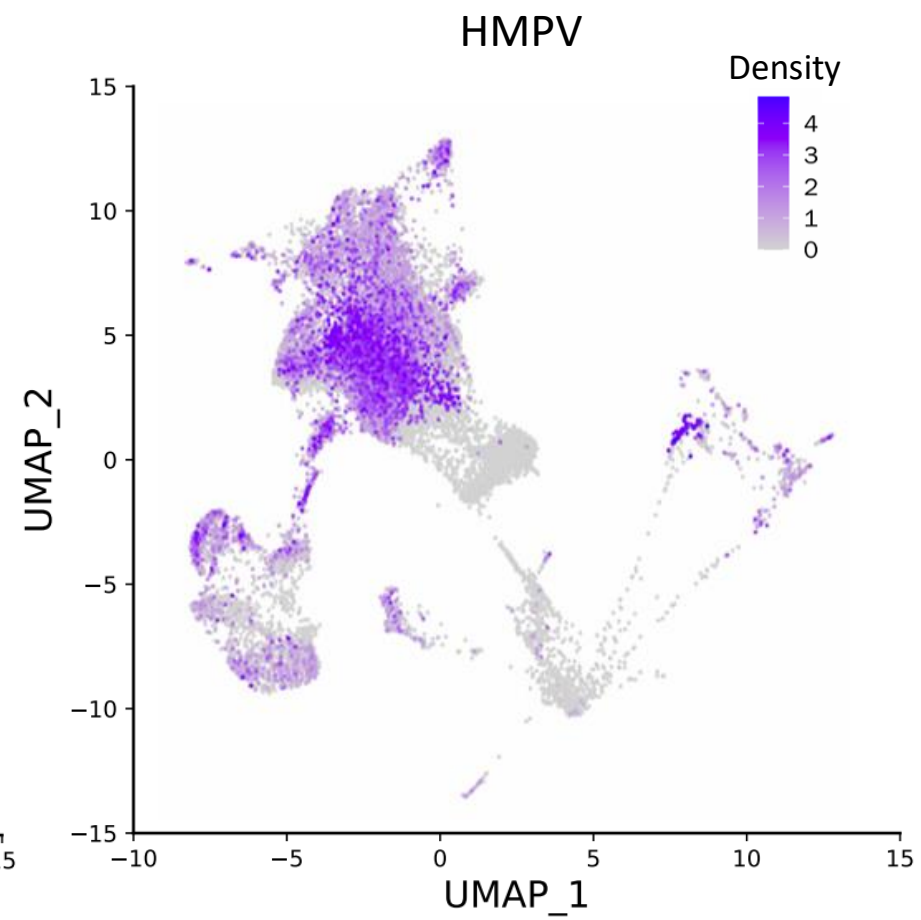

Supplement: qzad007_Supplementary_Data [file qzad007_supplementary_data.zip › Figure S2.pdf]

**A**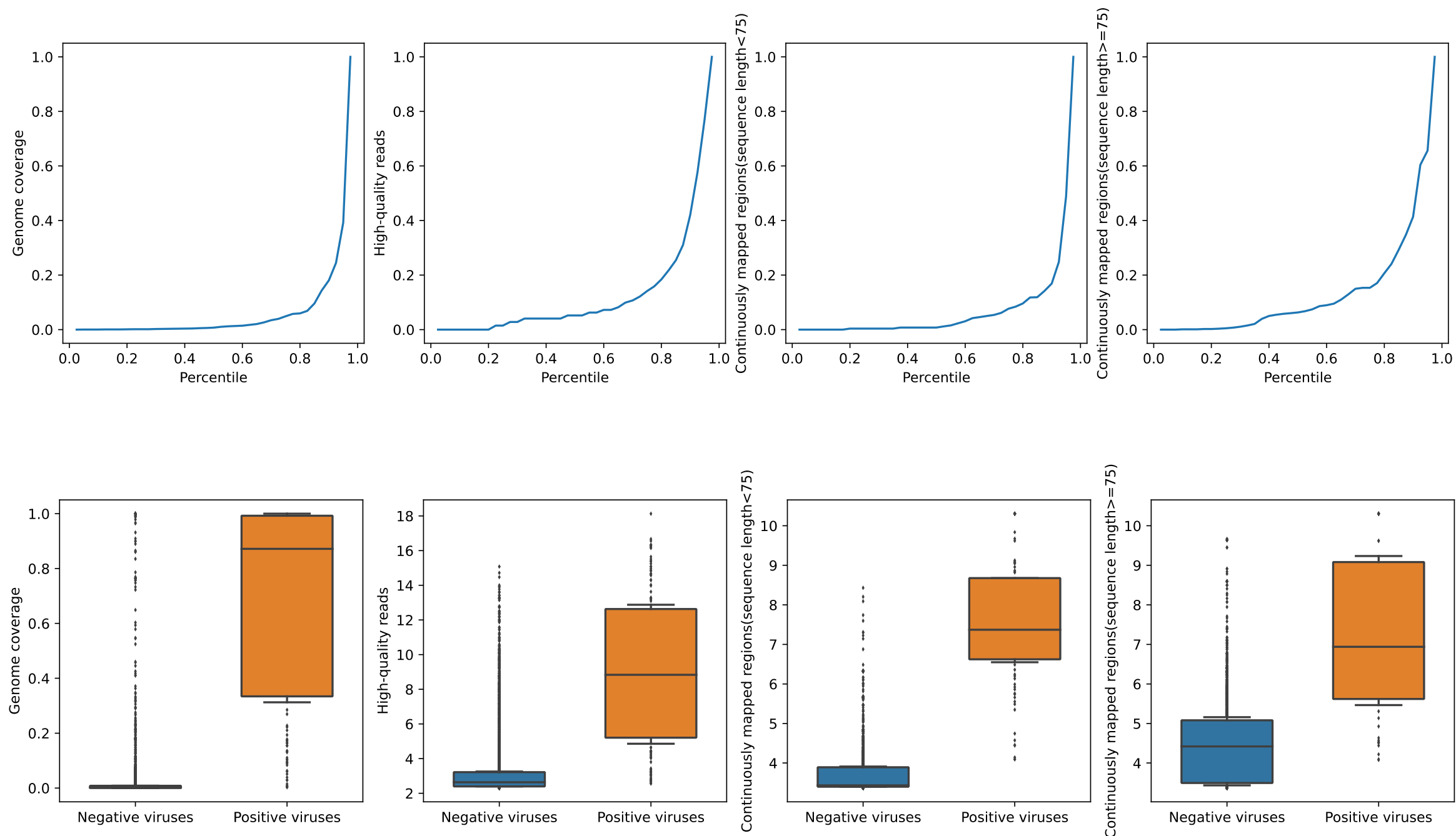**B**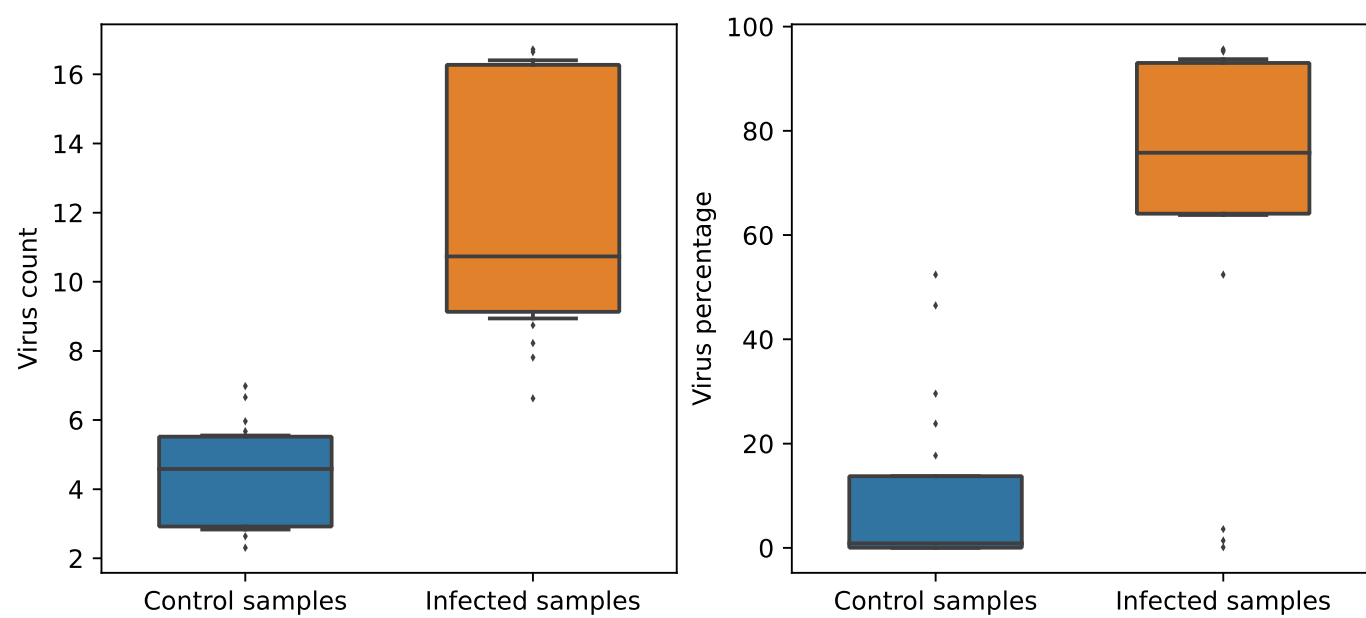

Supplement: qzad007_Supplementary_Data [file qzad007_supplementary_data.zip › Figure S1.pdf]
